# Mindfulness-Based Art Interventions for Students: A Meta-Analysis Review of the Effect on Anxiety

**DOI:** 10.3390/bs15081078

**Published:** 2025-08-07

**Authors:** Zhihui Zhu, Lin Xiao, Nor Aniza Ahmad, Samsilah Roslan, Nur Aimi Nasuha Burhanuddin, Jianping Gao, Cuihua Huang

**Affiliations:** 1 Faculty of Educational Studies, Universiti Putra Malaysia (UPM), Serdang 43400, Malaysia; 2 Faculty of Teacher Education, Baise University, Baise 533001, China

**Keywords:** mindfulness-based art interventions (MBAIs), students, anxiety, meta-analysis

## Abstract

Anxiety has become an important issue affecting students’ mental health. There is some evidence that mindfulness-based art interventions (MBAIs) can reduce students’ anxiety symptoms. However, some studies have shown the opposite view. Therefore, it is necessary to explore whether MBAIs are effective in alleviating students’ anxiety. In this meta-analysis, we chose 17 articles that met the criteria for inclusion, involving a total of 1548 participants, to figure out how big an impact the interventions had on student anxiety as a whole. The results show that MBAIs can reduce students’ anxiety (g = −0.387, *p* = 0.000). The effect size varies based on different moderators, including learning stage, sample size, intervention type, research design, measuring instrument, and intervention duration. Intervention type, research design, and measuring instrument are significant moderators. Specifically, the mindfulness-based art intervention (MBAI) showed stronger effects than the mandala coloring activity. Single-group experimental designs showed significantly higher effect sizes than studies that included a control group, and studies that used other measurement instruments had significantly higher effect sizes than those that used the State–Trait Anxiety Inventory. On this basis, the researchers put forward specific suggestions based on MBAIs to alleviate the anxiety of students from different educational backgrounds. However, due to the nascent nature of this field, the number of included articles is relatively small. The effectiveness of the research needs further testing.

## 1. Introduction

Anxiety is a negative emotion characterized by dread and apprehension for threatening but formless events in the future (Öhman, 2008). If the experience of anxiety lasts for a long time (usually six months or more), it can develop into an anxiety disorder (Craske et al., 2011). Anxiety disorder has been regarded as a leading mental burden all around the world (Collaborators, 2022; Yang et al., 2021). Until 2019, the World Health Organization reported that 301 million people worldwide suffered from anxiety disorder, with 58 million of them being children and adolescents (Collaborators, 2022). A meta-analysis of global anxiety disorder in about 204 countries and territories showed an extra 76.2 million instances increased massively in 2020, representing a 25.6% increase compared to previous years (Santomauro et al., 2021).

Mental health experts widely recognize student anxiety as a prevalent and urgent issue. A meta-analysis, including a large sample of students, revealed a considerable increase in anxiety levels since the 1950s (Twenge, 2000). Another meta-analysis of 203,678 Chinese students showed that anxiety levels reached 24% during the COVID-19 pandemic, and middle school children had an increased susceptibility to anxiety (Zhang et al., 2021). Previous studies have drawn a conclusion that chronic anxiety experience seriously affects students’ academic performance, social skills, and mental well-being (Brumariu et al., 2023; Dias Lopes et al., 2020; Pearcey et al., 2021; Russell & Topham, 2012).

The concept of mindfulness originates from ancient Buddhist scriptures over 2000 years ago (Rapgay & Bystrisky, 2009). In recent years, mindfulness has gained widespread popularity in various secular settings, including psychology, healthcare, and education. In 1979, American psychologist Kabat-Zinn introduced mindfulness into clinical psychology, defining it as the awareness that emerges through paying attention on purpose, in the present moment, and non-judgmentally to the unfolding of experience moment to moment (Kabat-Zinn, 2003). Studies have shown that mindfulness can effectively alleviate the anxiety levels of students and that the level of mindfulness is negatively correlated with anxiety—that is, the higher the level of mindfulness, the lower the level of anxiety among students (Lal & Vinod Kumar, 2024; Yılmazer et al., 2024).

According to Schuman-Olivier et al., Jon Kabat-Zinn created the first mindfulness-based intervention (MBI), known as mindfulness-based stress reduction (MBSR) (Schuman-Olivier et al., 2020). Initially, Kabat-Zinn designed MBSR as an 8-week structured program for patients to alleviate stress and pain in clinical practice (Kabat-Zinn, 2013b). It integrated mindfulness meditation techniques with elements from various contemplative traditions, including Buddhist insight meditation and yoga practices (Kabat-Zinn, 2013a). Existing findings indicated significant benefits for people with chronic pain, cancer, anxiety disorders, depression, and stress across many settings (Garmon et al., 2014; Hilton et al., 2017; Hofmann et al., 2010; Ledesma & Kumano, 2009; Schanche et al., 2020). With the promotion of MBSR, this intervention has been widely used in the general population and in other fields, including education. Previous research has shown that the intervention could not only effectively relieve stress, anxiety, depression, and other psychiatric problems but also improve academic attainment and well-being in different school settings (Baumgartner et al., 2023; Bennett & Dorjee, 2016; Gouda et al., 2016; Lensen et al., 2021).

Starting with the MBSR program, mindfulness-based interventions (MBIs) have gained widespread popularity in healthcare, psychology, and education, including Mindfulness-Based Cognitive Therapy (MBCT), Acceptance and Commitment Therapy (ACT), Dialectical Behavioral Therapy (DBT), Mindfulness-Based Relapse Prevention (MBRP), Mindful Self-Compassion (MSC) and Mindfulness-Based Art Therapy (MBAT). MBIs have shown efficacy in reducing symptoms of mental problems (such as anxiety, depression, and stress) and improving overall well-being. A review shows MBIs in schools identified enhancements in various aspects of well-being, cognitive performance, and resilience to stress among children and adolescents (Camacho, 2025).

Although MBIs have been shown to have positive effects on anxiety, some researchers are aware that traditional MBIs may face some limitations in school settings, especially for children who have difficulty staying calm or paying attention (Coholic, 2011). Coholic noted that many children had trouble keeping their eyes closed, which interrupted the process and made it difficult for them to stay physically motionless during traditional mindfulness therapies (Coholic, 2011). A meta-analysis examined the effects of MBIs on student stress, anxiety, and depression in schools, revealing that MBIs produced significant relief in adolescents’ stress in a school setting but not in depression and anxiety (Fulambarkar et al., 2023).

Mindfulness-based art interventions (MBAIs) are innovative therapeutic approaches that integrate mindfulness concepts with the expressive and creative processes of art therapy. At present, there is no unified operating standard and process for MBAIs. Different scholars have tried different activities to enhance the intervention effect. Bokoch and Hass-Cohen designed a School-Based Mindfulness and Art Therapy Group Program. This project is mainly aimed at children. It includes a total of eight weeks of activities. Each week’s activities include meditation practice (such as glitter jar meditation, breathing meditation, body scan meditation, mountain meditation, loving and kindness meditation) and art activities (such as playing with clay, drawing pictures, etc.) (Bokoch & Hass-Cohen, 2021). Mandala coloring is one common MBAI, referring to coloring mandala patterns so that participants can focus on the present moment (Mantzios & Giannou, 2018; Noor et al., 2017; Sari Ozturk & Kilicarslan Toruner, 2022).

Those interventions utilize the benefits of mindfulness and art to enhance results in reducing anxiety. Research has examined the efficacy of MBAIs in relieving anxiety in students. Experimental research involving 165 primary school pupils demonstrated the efficacy of a mindfulness-based art intervention (MBAI) in decreasing anxiety levels (Léger-Goodes et al., 2023). MBAIs have also demonstrated advantages in reducing anxiety among university students (Beerse et al., 2020). However, some other studies presented conflicting views. For instance, Carsley et al. utilized an experimental design to compare MBAIs with traditional coloring activities on students’ test anxiety, and the study did not demonstrate the superiority of mindfulness-based mandala drawing in improving mental health symptoms in children (Carsley et al., 2018). These research differences suggest that it is necessary to systematically integrate and analyze the existing literature to clarify the intervention effect of MBAIs on student anxiety. Therefore, it is essential to conduct a meta-analysis study to investigate the impact of MBAIs on students’ anxiety. The research questions of this study include the following:

RQ1: What is the overall effect size of MBAIs on students’ anxiety?

RQ2: What are the possible moderator variables significantly influencing this effect?

Based on the research questions, the research aims to examine the effect of MBAIs on students’ anxiety. This study will also explore possible moderators, which may have an impact on study outcomes, such as participant characteristics (e.g., learning stage), intervention characteristics (e.g., duration, types), and study design (e.g., sample size, research design, measuring instrument).

## 2. Methodology

### 2.1. Search Strategy

The search process ended on 10 May 2025. The databases searched in this study included EBSCOhost, Embase, ERIC, PubMed, Scopus, SpringerLink, and Google Scholar. Each database was searched by title and abstract. The search terms contain three parts: mindfulness-based art interventions (e.g., mindful* OR meditat* AND art OR drawing OR coloring OR painting AND intervention OR training OR program OR therapy OR treatment); anxiety (e.g., anxiety OR “mental problem”); students (e.g., student OR child* OR youth OR adolescen*). Boolean operators (AND, OR) and truncation (*) were applied to maximize sensitivity. As an example, the full search strategy used for PubMed was: ((((((mindful*[Title/Abstract] OR meditat*[Title/Abstract])) AND (art[Title/Abstract] OR drawing[Title/Abstract] OR coloring[Title/Abstract] OR painting[Title/Abstract])) AND (intervention*[Title/Abstract] OR training[Title/Abstract] OR program[Title/Abstract] OR therapy[Title/Abstract] OR treatment[Title/Abstract])) AND (anxiety[Title/Abstract] OR “mental problem”[Title/Abstract])) AND (student*[Title/Abstract] OR child*[Title/Abstract] OR youth[Title/Abstract] OR adolescen*[Title/Abstract])).

### 2.2. Eligibility Criteria

This study used the PICOS framework (population, intervention, comparison, outcome, and study design) to determine eligibility. This framework is from the Cochrane Handbook for Systematic Reviews of Interventions (see Table 1). The eligibility and exclusion criteria include seven aspects. (1) The population of this study is school students with anxiety in different educational environments. This study excludes populations with anxiety outside of school. (2) This study primarily focuses on MBAIs, excluding only mindfulness or art interventions. (3) Comparison was conducted without the use of MBAIs. (4) This study includes studies on the effects of MBAIs on students’ anxiety, excluding studies on the effects of MBAIs on other psychological problems. (5) This study includes one-group pre–post-test, quasi-experiment, and randomized controlled trial (RCT) designs. Excluded studies contained correlational studies, surveys, and review studies. (6) Studies must report sufficient data to calculate effect size (such as mean, standard deviation, sample size, t-value, *p*-value), and studies that cannot obtain the above data will be excluded. (7) Only studies published in English are suitable for inclusion. Publications in other languages are excluded.

### 2.3. Study Selection

First, two searchers screened all the databases with search technology at the same time. Then, we removed all the duplicate articles using the EndNote 9.0 software. Two autonomous investigators (Zhu and Xiao) examined the titles and abstracts of the studies. We obtained studies that met the inclusion criteria for a comprehensive evaluation of the full text. The two reviewers read full-text articles carefully and discussed the differences. The disagreements were solved with the attendance of the third investigator (Gao).

### 2.4. Data Extraction and Quality Assessment

After screening the literature, we used the QualSyst quality assessment tool (Kmet et al., 2004) to assess the quality of the included studies. The tool contains 14 assessment items (see Table 2), each of which is scored according to “yes (2 points), partly (1 point), no (0 point)”. Items that are not applicable to a specific study design are marked as NA and excluded from the total score calculation. The higher the total score, the higher the quality of the study. The quality level of the study is divided into high quality (score ≥ 75%), medium quality (55–75%), and low quality (<55%).

During the scoring process, two researchers (Zhu and Xiao) completed the scoring independently. When there was a disagreement, it was resolved through discussion. If there was still a disagreement, it was arbitrated by a third researcher (Gao). To evaluate the consistency between the raters, we calculated Cohen’s kappa coefficient to test the consistency of the scoring results.

### 2.5. Data Analysis

Effect sizes were determined using mean and standard deviation estimates. When means and standard deviations were not accessible, t and *p* statistics were used to calculate effect sizes. Each outcome variable had a single effect size when numerous instruments were used to evaluate the same outcome (Thalheimer & Cook, 2002). Each study’s effect size was calculated using Hedges’ g and included the associated *p*-value and its 95% confidence interval (CI). The selection of Hedges’ g was based on its reduced bias for predicting effect sizes in small sample sizes (Hedges & Olkin, 2014). Data for outcome variables were combined for meta-analysis using the random-effects model. Cohen’s paradigm of effect sizes, with values of 0.2 for small, 0.5 for medium, and 0.8 for large, was used to evaluate the effect size.

The heterogeneity of the effect sizes was evaluated using the Cochrane Q and I^2^ statistics. The Q-test assesses heterogeneity caused by sampling error, although it may lack the sensitivity to identify genuine heterogeneity. The I^2^ statistic was used to assess whether the differences between trials were caused by heterogeneity rather than random sampling error (Higgins & Thompson, 2002). The I^2^ number ranges from 0 to 100%, representing levels of heterogeneity as follows: 0% for no heterogeneity, 25% for low heterogeneity, 50% for medium heterogeneity, and 75% for high heterogeneity. In order to evaluate the robustness of the combined effect value, this study conducted a sensitivity analysis, using the leave-one-out method to recalculate the combined effect value to test whether a single study has a significant impact on the overall results.

This study used funnel plots, Egger regression tests, and fail-safe N to comprehensively evaluate publication bias. Specifically, the funnel plot plots the relationship between the effect size of each study and its standard error. If the effect size is symmetrically distributed around the mean, it indicates that there is no obvious bias. On the contrary, a skewed distribution suggests that there may be a lack of small sample studies (Light & Pillemer, 1984). However, there is obvious subjectivity in using funnel plots to judge publication bias. Therefore, we used Egger regression tests to further statistically test the asymmetry of the funnel plot to reduce the limitations of subjective judgment. In addition, the fail-safe N estimates how many zero-effect studies need to be added to make the overall effect value lose significance, thereby quantifying the impact of potential missing studies on the results (Lipsey & Wilson, 2001).

## 3. Results

### 3.1. Study Selection Procedure

**Figure 1:**
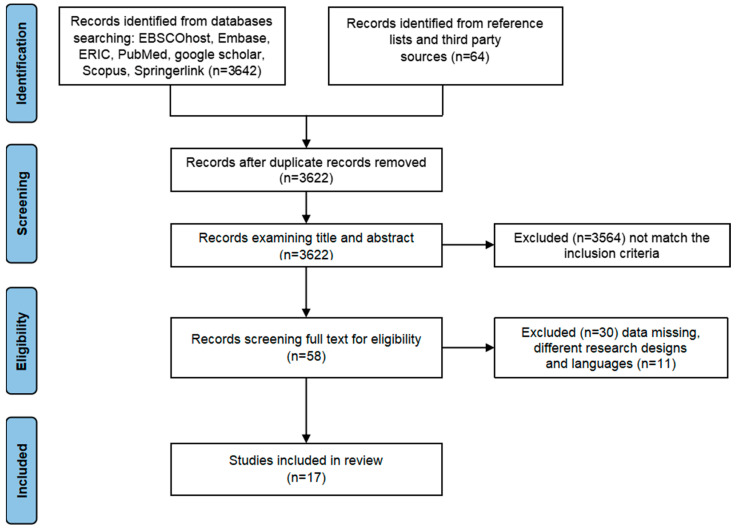
The flow diagram of study selection procedure.

There are 3706 articles according to all databases, including EBSCOhost, Embase, ERIC, PubMed, Scopus, Springerlink, and Google Scholar. Further, 84 articles were removed because of duplication, and 3622 articles remained for further screening. After examining the title and abstract, 3564 articles were eliminated because they did not match the inclusion criteria. Then, 58 full-text articles were investigated, and 41 articles were excluded because of missing data, different research designs, and languages. Finally, 17 articles met all characteristics required in this study (Figure 1).

### 3.2. Study Quality Assessment

Two researchers independently scored the 17 included articles, and the inter-rater reliability was satisfactory (Cohen’s kappa = 0.84). According to the QualSyst tool, 14 studies were classified as high quality, and 3 studies were classified as medium quality. The quality scoring results are detailed in Table 2.

### 3.3. Study Characteristics

A total of 17 studies (n = 1548 participants) met the inclusion criteria. The sample size of each study ranged from 20 to 193. The participants covered three educational levels: primary school (n = 4), middle school (n = 1), and university (n = 12). All studies evaluated a mindfulness-based arts intervention, the most common of which were mandala coloring (n = 14) and MBAI (n = 3). The duration of the intervention ranged from 1 to 10 weeks, with the majority of them being short-term interventions (n = 11). Regarding study design, 10 were randomized controlled trials, 5 were quasi-experimental, and 2 used a one-group experimental design. The study outcomes were mainly measured using the State–Trait Anxiety Inventory (n = 12), while the remaining five studies used other anxiety scales. Detailed information on study characteristics is provided in Table 3.

### 3.4. Effect Size and Homogeneity Testing

**Figure 2:**
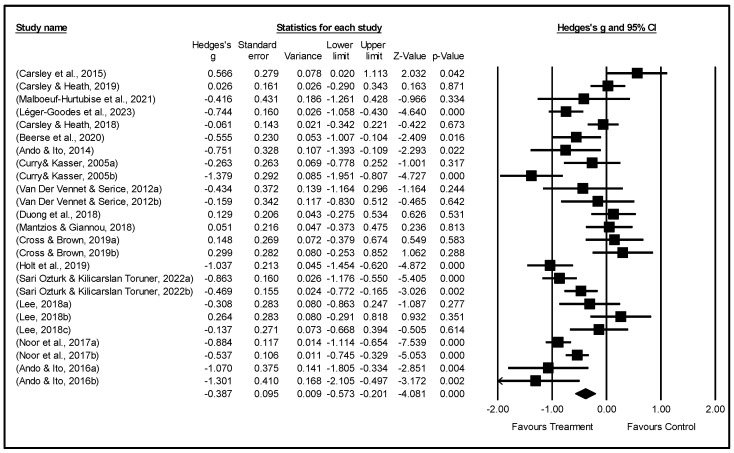
Forest plot for the random-effects model.

Due to differences in samples, measurements, and designs among studies, such variations may lead to heterogeneity between studies. In such circumstances, using a random-effects model can better capture this heterogeneity, thereby enhancing the accuracy and reliability of meta-analyses (Viechtbauer, 2007). Therefore, this study employed a random-effects model to examine the overall effect size and heterogeneity (see Table 4). When combining the 25 effect sizes from all studies, the overall estimated effect size was −0.387. This indicates that MBAIs reduced students’ standardized anxiety scores by 0.387 standard deviations. Although this effect size falls within the range of a small to moderate effect, it holds statistical significance (z = −4.081, *p* = 0.000). The 95% CI for the average effect size is (−0.573, −0.201), suggesting that in 95% of cases, the true average effect size of MBAIs will fall between −0.573 and −0.201. The forest plot provides a more intuitive display of the analysis results. It describes a series of estimates and their corresponding 95% CI (see Figure 2), with each study’s effect size presented in square form. The center of the square represents the estimated effect size of the study, while the horizontal line extending from the square represents the 95% CI. The plot demonstrates wider CI and inconsistent response rates, highlighting the heterogeneity among the selected studies.

The heterogeneity test yielded a Q statistic of 113.994 and a *p*-value less than 0.001, indicating significant heterogeneity in effect sizes among studies. The statistical estimation of the I^2^ value is 78.946%, with the variance of the average effect size being 0.161 (T^2^ statistic) and the standard deviation being 0.401 (T statistic). This means that 78.946% of the total variation between studies is due to real differences in effect size, rather than sampling error. This variability may be attributed to various factors such as study designs, sample characteristics, etc. Therefore, it is necessary to further explore potential moderating factors that may influence effect size to explain this variability and ensure a more accurate and comprehensive interpretation and inference of the study results.

### 3.5. Sensitivity Analysis

Sensitivity analysis showed that after removing a single study one by one, the direction and significance of the combined effect size did not change significantly, and the effect size ranged from g = −0.424 (95% CI −0.605 to −0.243) to g = −0.350 (95% CI −0.533 to −0.166). This shows that the results of this study are relatively robust and are not significantly affected by a single study.

### 3.6. Publication Bias

**Figure 3:**
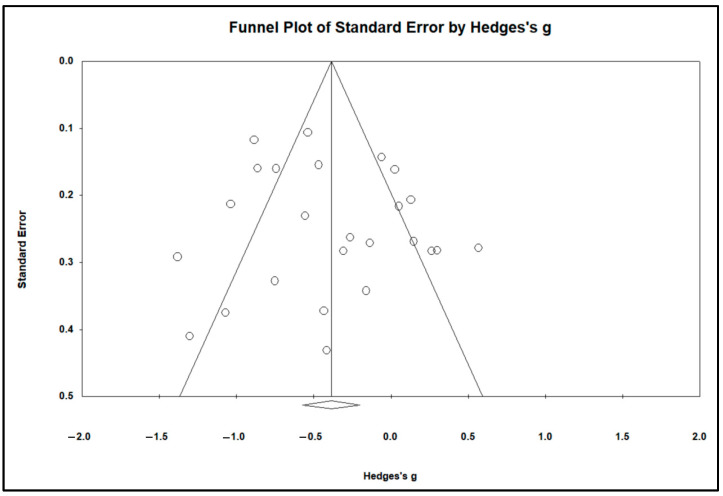
Funnel plot of the overall mean effect size analysis.

This study employed funnel plots, Egger’s test, and fail-safe N to assess publication bias. From the funnel plot (see Figure 3), it can be observed that the majority of effect sizes fall within the funnel’s effective area, with a distribution on both sides of the mean effect size, showing no evident asymmetry. Egger’s regression test yielded a *p*-value of 0.373, indicating no signs of publication bias. Moreover, the classic fail-safe N was 526, a value significantly larger than the threshold of 135 (z = −9.199, *p* = 0.000; 5k + 10 = 135; Card, 2015). Therefore, based on the funnel plot and related statistical data, we conclude that the impact of publication bias is not excessively magnified.

### 3.7. Subgroup Analyses

To explore potential reasons for significant heterogeneity, this study conducted moderator analysis using a random-effects model, including learning stage, intervention type, research design, measuring instrument, and research duration.

#### 3.7.1. Learning Stage

The numerical results in Table 5 show that the effect sizes of students at different stages of learning did not reach a significant level (Q_B_ = 1.914, *p* = 0.167, *p* > 0.05). However, compared to the K-12 stage (g = 0.128, 95% CI −0.545 to 0.288, *p* = 0.546), the university stage may be associated with higher effect sizes (g = −0.445, 95% CI −0.657 to −0.254, *p* = 0.000).

#### 3.7.2. Intervention Type

The studies included in the meta-analysis employed various interventions to alleviate student anxiety, including mandala coloring and MBAI. Subgroup analysis revealed significant differences in effect sizes among different intervention types (Q_B_ = 6.602, *p* = 0.010, *p* < 0.05) (see Table 5). The effect of MBAI on students’ anxiety (g = −0.803, 95% CI −1.119 to −0.487, *p* = 0.000) reached a large effect size, which was significantly higher than mandala coloring (g = −0.312, 95% CI −0.513 to −0.111, *p* = 0.002).

#### 3.7.3. Research Design

The anxiety intervention studies utilized different types of research designs, including one-group experiments and control group experiments. Subgroup analysis revealed significant differences in effect sizes among different research designs (Q_B_ = 7.977, *p* = 0.005, *p* < 0.05) (see Table 5), indicating that research design serves as a potential moderator between experimental interventions and anxiety. Specifically, the effect of one-group experiments on students’ anxiety (g = −0.816, 95% CI −1.117 to −0.516, *p* = 0.000) was significantly higher than that of control group experiments (g = −0.293, 95% CI −0.497 to −0.089, *p* = 0.005).

#### 3.7.4. Intervention Duration

As can be seen from Table 5, the effect sizes of different intervention durations did not reach a significant level (Q_B_ = 1.254, *p* = 0.263, *p* > 0.05). However, compared with one week (g = −0.330, 95% CI −0.570 to −0.090, *p* = 0.007), experiments lasting more than one week may have a higher effect size (g = −0.533, 95% CI −0.797 to −0.270, *p* = 0.000).

#### 3.7.5. Measuring Instrument

The results of subgroup analysis showed that there were significant differences in the effect sizes between different anxiety measurement tools (QB = 9.154, *p* = 0.002) (see Table 5). Among them, the studies using other measurement tools had a greater intervention effect on student anxiety (g = −0.748, 95% CI −0.960 to −0.536, *p* = 0.572), and its effect size was significantly higher than that of the studies using the State–Trait Anxiety Inventory (g = −0.282, 95% CI −0.497 to −0.066, *p* = 0.001).

### 3.8. Meta-Regression

To determine whether sample size significantly predicts effect sizes using different models, this study conducted univariate meta-regression analysis (Table 6). The results showed that, although the sample size had a slight negative impact on the research results, its significance was not significant (estimate = −0.0002, SE = 0.002, QM = 0.010, *p* = 0.903, *p* > 0.05). This shows that although the effect size of MBAIs on students’ anxiety level will decrease slightly with an increase in sample size, when the sample size is used as a predictor, its explanatory power for the research results is relatively weak.

## 4. Discussion

This meta-analysis presents an overall examination of the effects of MBAIs on students’ anxiety. The result shows that MBAIs could reduce the symptoms of students’ anxiety significantly. In addition, the effect varies based on different intervention moderators, including learning stage, intervention type, research design, intervention duration, measuring instrument, and sample size.

### 4.1. The Relationship Between MBAIs and Students’ Anxiety

Numerous scholars have already developed diverse MBAIs and reported different outcomes on the relationship between these interventions and student anxiety. However, there has been no comprehensive study to determine whether they actually reduce anxiety so far. To address this research gap, this meta-analysis systematically assessed the relationship between MBAIs and student anxiety by examining 17 included studies with about 1548 participants. The results showed a significant negative effect between the MBAIs and student anxiety overall (g = −0.387). According to Cohen’s criteria, this effect size is small to medium, which suggests that MBAIs have potential benefits in alleviating student anxiety, which is consistent with the view that MBAIs can relieve student anxiety (Curry & Kasser, 2005; Léger-Goodes et al., 2023; Malboeuf-Hurtubise et al., 2021). However, whether this anxiety-reducing effect is clinically significant and whether it can be translated into substantial improvements in learning performance in real educational situations still need further verification. In particular, due to the high heterogeneity of the included studies, the effect size may be affected by bias.

### 4.2. Moderating Effects

#### 4.2.1. Learning Stage

The subgroup analysis in Table 5 indicated that the learning stage had no significant effect on the overall effect size (*p* = 0.167, *p* > 0.05). However, the MBAIs had a significant effect on college students’ anxiety and achieved a medium effect size (g = −0.445, *p* = 0.000). Previous research also confirmed this finding. As Galla pointed out, the reason why MBIs can be extended to adolescents in schools is largely due to their success in adults (Galla, 2024). Adolescents may get limited benefits from MBIs due to their insufficient meta-cognitive capacities necessary for mindfulness (Gould et al., 2012). In addition, it may also be because the research on MBAIs is still in its infancy, and there are not sufficient studies regarding primary and secondary school students as study groups. Subsequent researchers should fully grasp this research gap in order to more completely and comprehensively understand the effects of mindfulness-based artistic sense on students at different learning stages.

#### 4.2.2. Intervention Type

When it comes to intervention type, Table 5 shows that it can influence the effectiveness of MBAIs on students’ anxiety (*p* = 0.010, *p* < 0.05). Compared with mandala coloring, MBAI reached the higher effect size (g = −0.803). The results of this study are consistent with previous studies on children’s mindfulness. Galla contended that motivation constrained the effectiveness of universal school-based mindfulness intervention for adolescents (Galla, 2024). The MBAI has more diverse forms and more intriguing activities (e.g., yoga, clay sculpture, and collage), which can attract students’ attention and interest (Ando & Ito, 2014, 2016; Beerse et al., 2020; Cheshure et al., 2023). However, mandala coloring required students to concentrate on their activities and conduct them independently (Carsley & Heath, 2019; Van Der Vennet & Serice, 2012). It is difficult and unattractive for some students. It is also possible that this is due to the length of the intervention. It should be noted that the heterogeneity of the mandala coloring group was high (I^2^ = 80.99%), indicating that there were large differences between the studies within the group, which may affect the stability of the effect size estimate. The results of the sensitivity analysis showed that after eliminating each study one by one, the significance of the combined effect value did not change significantly, indicating that the overall results were relatively robust. In contrast, the heterogeneity of the MBAI group was extremely low (I^2^ = 4.99%), indicating that the results of the studies within the group were highly consistent. This may be due to the high degree of structure of the MBAI intervention program and the strong professionalism of the facilitators. In addition, the number of studies in the MBAI group was small (k = 4), and more high-quality studies are still needed to verify its effect in the future.

#### 4.2.3. Research Design

This study showed that different types of research designs had a significant impact on the effectiveness of MBAIs in relieving student anxiety (*p* = 0.005, *p* < 0.05). Specifically, in one-group experiment designs, the average effect size of the intervention was large (g = −0.816), while in experiments with a control group, the effect size was relatively small (g = −0.293). This may be due to the degree of control over irrelevant variables. One-group experiment designs are easily affected by irrelevant factors due to the lack of control conditions. In comparison, the control group experimental design is ideal for maximizing internal validity and controlling the highest degree of irrelevant variables (Ross & Morrison, 2013). Therefore, future studies should give priority to the control group experimental designs and strictly control irrelevant factors to obtain more robust and generalizable evidence.

#### 4.2.4. Intervention Duration

Table 4 shows that the intervention duration did not significantly affect students’ anxiety (*p* = 0.263, *p* > 0.05). This conclusion is consistent with the results of some previous studies, which have shown that long-term and short-term mindfulness practices have no significant difference in anxiety (Berghoff et al., 2017; Strohmaier et al., 2021). However, the effect of MBAIs with an intervention time of more than one week (g = −0.533) on alleviating students’ anxiety was better than that of a short-term intervention with an intervention time of no more than one week (g = −0.330). It shows that the effect of mindfulness practice depends on long-term practice accumulation. This supports some previous theoretical studies. Based on mode of mind theory, it proposes that continuous mindfulness practice is needed to change mental models and obtain a positive psychological state (Williams, 2008). According to the theory of neuroplasticity, continuous mindfulness practice can gradually reshape the brain structure and regulate the functional connection with related brain areas (such as the amygdala and prefrontal cortex) (Gotink et al., 2016; Vago & Silbersweig, 2012). Therefore, in future studies, the intervention duration can be appropriately extended to ensure the stability and durability of the intervention effect.

#### 4.2.5. Measuring Instrument

This study showed that the intervention effects under different measurement tools were different. The combined effect size of studies using the STAI (State–Trait Anxiety Inventory) (k = 19) was small (g = −0.282, 95% CI −0.497 to −0.066, *p* < 0.001). In contrast, the combined effect size of studies using other tools (k = 6) was large (g = −0.748, 95% CI −0.960 to −0.536, *p* < 0.001). This difference may be related to the sensitivity and focus of the measurement tools. The state anxiety scale in STAI reflects the anxiety level of an individual at a specific time point and is easily affected by external situations, which may lead to biased effect size estimates (Spielberger, 1983). In contrast, studies using other tools (such as GAD-7) tend to assess general anxiety symptoms (Spitzer et al., 2006) and may be more sensitive to the effects of mindfulness-based art interventions. In addition, further analysis found that the sample size of studies using other scales was generally small, and the existence of small sample bias may also lead to an overestimation of the effect size (Egger et al., 1997). This finding suggests that when interpreting the effects of mindfulness-based art interventions, it is necessary to fully consider the construct differences in the measurement tools and the potential bias of small samples.

#### 4.2.6. Sample Size

Although the results in Table 5 show that differences in sample size had no significant difference in the impact of MBAIs on student anxiety (*p* = 0.903, *p* > 0.05), the effect size of MBAIs on students’ anxiety level will decrease slightly with an increase in sample size. This may be related to the operational requirements of mindfulness interventions. In the operation instruction, participants are suggested to close their eyes and concentrate on their breathing and body feelings (Grecucci et al., 2015). Activities with a small sample size may be best for students to maintain deep focus and for teachers to provide personalized guidance and feedback.

## 5. Research Limitations and Further Study

Although this meta-analysis effectively provides strong support for the effect of MBAIs on reducing student anxiety, there are still some limitations in terms of the inclusion of the literature and mediator analysis.

Since MBAIs are still in their early stages, this study can only incorporate a limited amount of the existing literature. The sample size of the included studies is generally small, which may affect the stability of the effect size estimation and the statistical power of the meta-regression analysis. In the future, researchers should include more large-sample high-quality studies to obtain more reliable results.

This study only analyzed six moderating factors: learning stage, intervention type, research design, measuring instrument, and research duration. It failed to conduct moderating effect analysis on variables, such as age, geographic region, facilitator type, and delivery context. This is mainly attributed to the limited number of studies on relevant variables or the lack of detailed reports in the original studies, which limits the possibility of further exploring the differences in intervention effects in different contexts. Future studies should strengthen the collection and reporting of information on such variables to support more comprehensive moderating effect analysis.

Although the Comprehensive Meta-Analysis Software (CMA, version 3.7) used in this study can perform subgroup analysis and univariate regression analysis, it cannot simultaneously explore the potential interactions between multiple moderating variables and perform multiple comparison corrections, which limits the in-depth revelation of the complex mechanisms of the intervention effect. Future studies can use software such as Stata or R for interaction analysis and multiple comparison corrections to improve the robustness and generalizability of the research results.

## 6. Conclusions

This meta-analysis systematically evaluated the potential of MBAIs in reducing students’ anxiety levels. The results showed that the intervention was statistically significant (g = −0.387, *p* = 0.000), and the effect size was small to moderate, suggesting that MBAIs have potential application value in reducing students’ anxiety.

Subgroup analysis further showed that intervention type, research design, and measurement tools were significant moderating factors. Specifically, MBAI intervention was more effective than mandala coloring activities, but given the limited number of related studies, its effectiveness still needs to be further verified. There were significant differences in the effect sizes reported in studies using different measurement tools, suggesting that the choice of measurement tools may affect the evaluation of intervention effects. Future studies can develop more appropriate measurement tools according to the type of anxiety to improve the accuracy of research conclusions. It is worth noting that the single-group experimental design showed a significantly higher effect size than studies with a control group, which may not mean that its intervention effect is better but may be due to the estimation bias caused by the lack of control group comparison. Therefore, future studies should give priority to the randomized controlled experimental design and strengthen the rigor of research methods to improve the internal validity of intervention effect evaluation. In addition, although variables such as learning stage, intervention duration, and sample size did not show significant moderating effects, these factors may affect the intervention effect in specific contexts. Future studies can explore how these variables jointly modulate the intervention effect through multivariate meta-regression and interaction analysis and examine the differences in the role of education stage, cultural background, and geographical region in the intervention.

The results of this study provide preliminary evidence for the application of MBAIs in the field of education. It can be used as an auxiliary psychological intervention tool to help students manage anxiety, especially during periods of academic pressure. Educators may consider incorporating it into mental health courses, but attention should be paid to the standardization of intervention implementation and professional training of teachers. In addition, intervention programs suitable for different educational stages should be developed, such as designing simplified mindfulness art activities in primary and secondary schools and introducing more complex and in-depth exercises in university to match students’ cognitive development levels.

Policymakers should pay attention to the resource allocation and feasibility of interventions, such as providing relevant material resources, conducting teacher training, formulating implementation guidelines, and adjusting the intervention content in combination with cultural adaptability to ensure its effectiveness in different regions and cultural contexts.

## Figures and Tables

**Table 1:** The inclusion and exclusion criteria according to PICOS framework.

Parameter	Inclusion Criteria	Exclusion Criteria
Population	School students with anxiety in different educational environments	Population with anxiety outside of school
Intervention	Mindfulness based art interventions (MBAIs)	Mindfulness interventions only or art interventions only
Comparison	Other interventions reducing students’ anxiety	Mindfulness based art interventions (MBAIs)
Outcome	The effects of MBAIs on students’ anxiety	The effects of MBAIs on students’ other mental problems
Study design	One-group pre–post-test, quasi-experiment, and randomized controlled trial (RCT) designs	Correlational study, surveys, and reviews study
Study data	Studies must report sufficient data to calculate effect size (such as mean, standard deviation, sample size, t-value, *p*-value)	Studies that did not report key statistics and whose information could not be completed after contacting the corresponding author
Publication language	Published in English	Publications in other languages

**Table 2:** Quality assessment through QualSyst.

References	I	II	III	IV	V	VI	VII	VIII	IX	X	XI	XII	XIII	XIV	Rating
(Carsley et al., 2015)	2	2	2	2	2	0	0	2	1	2	2	1	2	2	Strong
(Carsley & Heath, 2019)	2	2	2	2	2	0	0	2	2	2	2	1	2	2	Strong
(Malboeuf-Hurtubise et al., 2021)	2	2	2	2	1	0	2	2	0	2	2	2	2	2	Strong
(Léger-Goodes et al., 2023)	2	2	2	1	1	0	2	2	2	2	2	2	2	2	Strong
(Carsley & Heath, 2018)	2	2	2	2	2	0	0	2	2	2	2	2	2	2	Strong
(Beerse et al., 2020)	2	2	2	1	2	0	0	2	2	2	2	1	2	2	Strong
(Ando & Ito, 2014)	2	1	2	2	1	0	0	2	1	2	2	1	2	2	Moderate
(Curry & Kasser, 2005)	2	2	2	1	2	0	0	2	1	2	2	1	2	2	Strong
(Van Der Vennet & Serice, 2012)	2	2	2	2	2	0	0	2	1	2	2	1	2	2	Strong
(Duong et al., 2018)	2	2	2	1	1	0	0	2	2	2	2	1	2	2	Strong
(Mantzios & Giannou, 2018)	2	2	2	2	2	0	2	2	2	2	2	0	2	2	Strong
(Cross & Brown, 2019)	2	2	2	2	1	0	0	2	1	2	2	1	2	2	Strong
(Holt et al., 2019)	2	2	2	2	2	0	0	2	2	2	2	2	2	2	Strong
(Sari Ozturk & Kilicarslan Toruner, 2022)	2	2	2	1	2	1	1	2	2	2	2	1	2	2	Strong
(Lee, 2018)	2	2	2	1	2	0	0	2	1	2	2	1	2	2	Strong
(Noor et al., 2017)	2	1	1	2	NA	NA	NA	2	2	2	2	0	2	2	Moderate
(Ando & Ito, 2016)	2	1	2	1	NA	NA	NA	2	0	2	2	1	2	2	Moderate

I. Question/objective sufficiently described? II. Study design evident and appropriate to answer study question? III. Method of subject/comparison group selection or source of information/input variables described and appropriate? IV. Subject (and comparison group, if applicable) characteristics sufficiently described? V. If random allocation to treatment group was possible, is it described? VI. If interventional and blinding of investigators was possible, was it reported? VII. If interventional and blinding of subjects was possible, was it reported? VIII. Outcome and (if applicable) exposure measure(s) well defined and robust to measurement/misclassification bias? Means of assessment reported? IX. Sample size appropriate? X. Analysis described and appropriate? XI. Some estimate of variance is reported for the main results? XII. Controlled for confounding? XIII. Results reported in sufficient detail? XIV. Conclusions supported by the results?

**Table 3:** Study characteristics of all the included studies.

No.	References	Participants	Intervention Types	Duration (Weeks)	Sample Size	Study Designs	Measuring Instrument
1	(Carsley et al., 2015)	Primary school students	Mandala coloring	1	52	RCT	STAI
2	(Carsley & Heath, 2019)	Primary school students	Mandala coloring	1	152	RCT	STAI
3	(Malboeuf-Hurtubise et al., 2021)	Primary school students	Mandala coloring	5	22	Quasi-experiment	Others
4	(Léger-Goodes et al., 2023)	Primary school students	Mandala coloring	10	165	Quasi-experiment	Others
5	(Carsley & Heath, 2018)	Middle school students	Mandala coloring	1	193	RCT	STAI
6	(Beerse et al., 2020)	University students	MBAI (mindfulness practice and intentional art making with earth-based clay)	5	77	RCT	Others
7	(Ando & Ito, 2014)	University students	MBAI (Mindfulness therapy included breathing, yoga, and body scan; Arts items using clay, collage, or drawing)	4	39	Quasi-experiment	Others
8	(Curry & Kasser, 2005)	University students	Mandala coloring	1	57	RCT	STAI
9	(Van Der Vennet & Serice, 2012)	University students	Mandala coloring	1	50	RCT	STAI
10	(Duong et al., 2018)	University students	Mandala coloring	5	93	Quasi-experiment	STAI
11	(Mantzios & Giannou, 2018)	University students	Mandala coloring	1	84	RCT	STAI
12	(Cross & Brown, 2019)	University students	Mandala coloring	1	76	Quasi-experiment	STAI
13	(Holt et al., 2019)	University students	Mandala coloring	1	99	RCT	STAI
14	(Sari Ozturk & Kilicarslan Toruner, 2022)	University students	Mandala coloring	3	170	RCT	STAI
15	(Lee, 2018)	University students	Mandala coloring	1	99	RCT	STAI
16	(Noor et al., 2017)	University students	Mandala coloring	1	100	One-group PR-PO	STAI
17	(Ando & Ito, 2016)	University students	MBAI (Mindfulness therapy included breathing, yoga, and body scan; Arts items using clay, collage, or drawing)	1	20	One-group PR-PO	Others

MBAI, mindfulness-based art intervention; STAI, State–Trait Anxiety Inventory; RCT, randomized controlled trial; PR, pre-test; PO, post-test.

**Table 4:** Summary of overall mean effect size and test for heterogeneity.

Effect Size and 95% CI			Heterogeneity Test			Tau-Squared			Test of Null (Two-Tailed)	
k	g	95% CI	Q	*p*	I^2^	Tau^2^	SE	Tau	Z	*p*
25	−0.387	[−0.573, −0.201]	113.994	0.000	78.946	0.161	0.070	0.401	−4.081	0.000

**Table 5:** Subgroup analysis results.

Moderator Variables	k	Hedges’ g	SE	95% CI	I^2^	Q_W_	*p*
**Learning stage**	**Q_B_ = 1.914 (*p* = 0.167)**						
K-12	5	−0.128	0.213	[−0.545, 0.288]	81.958	22.170	0.546
Higher education	20	−0.445	0.103	[−0.657, −0.254]	75.972	79.074	0.000
**Intervention type**	**Q_B_ = 6.602 (*p* = 0.010)**						
Mandala coloring	21	−0.312	0.103	[−0.513, −0.111]	80.986	105.187	0.002
MBAI	4	−0.803	0.161	[−1.119, −0.487]	4.994	3.158	0.000
**Research design**	**Q_B_ = 7.977 (*p* = 0.005)**						
One-group experiment	4	−0.816	0.153	[−1.117, −0.516]	61.490	7.790	0.000
Control-group experiment	21	−0.293	0.104	[−0.497, −0.089]	76.585	85.417	0.005
**Intervention duration**	**Q_B_ = 1.254 (*p* = 0.263)**						
≤1 week	18	−0.330	0.122	[−0.570, −0.090]	81.805	93.435	0.007
>1 week	7	−0.533	0.134	[−0.797, −0.270]	64.434	16.870	0.000
**Measuring instrument**	**Q_B_ = 9.154 (*p* = 0.002)**						
State-trait anxiety inventory	19	−0.282	0.110	[−0.497, −0.066]	82.104	100.582	0.000
Other instruments	6	−0.748	0.108	[−0.960, −0.536]	0.000	3.845	0.572

**Table 6:** Meta-regression analysis of continuous variables.

Variable	Estimate	SE	95% CI	Z	*p*	Q_M_	df
Simple size	−0.000	0.002	[−0.004, 0.003]	−0.120	0.903	0.010	1

## Data Availability

The raw data supporting the conclusions of this article will be made available by the authors on request.

## References

[B1-behavsci-15-01078] Ando M., Ito S. (2014). Potentiality of mindfulness art therapy short version on mood of healthy people. Health.

[B2-behavsci-15-01078] Ando M., Ito S. (2016). Changes in autonomic nervous system activity and mood of healthy people after mindfulness art therapy short version. Health.

[B3-behavsci-15-01078] Baumgartner P., Jennifer N., Schneider P., Tamera R. (2023). A randomized controlled trial of mindfulness-based stress reduction on academic resilience and performance in college students. Journal of American College Health.

[B4-behavsci-15-01078] Beerse M. E., Van Lith T., Stanwood G. (2020). Therapeutic psychological and biological responses to mindfulness-based art therapy. Stress and Health.

[B5-behavsci-15-01078] Bennett K., Dorjee D. (2016). The impact of a mindfulness-based stress reduction course (MBSR) on well-being and academic attainment of sixth-form students. Mindfulness.

[B6-behavsci-15-01078] Berghoff C. R., Wheeless L. E., Ritzert T. R., Wooley C. M., Forsyth J. P. (2017). Mindfulness meditation adherence in a college sample: Comparison of a 10-min versus 20-min 2-week daily practice. Mindfulness.

[B7-behavsci-15-01078] Bokoch R., Hass-Cohen N. (2021). Effectiveness of a school-based mindfulness and art therapy group program. Art Therapy.

[B8-behavsci-15-01078] Brumariu L. E., Waslin S. M., Gastelle M., Kochendorfer L. B., Kerns K. A. (2023). Anxiety, academic achievement, and academic self-concept: Meta-analytic syntheses of their relations across developmental periods. Development and Psychopathology.

[B9-behavsci-15-01078] Camacho C. R. (2025). Effectiveness of Mindfulness-Based Interventions in Education: A Review. Challenges of educational innovation in contemporary society.

[B10-behavsci-15-01078] Card N. A. (2015). Applied meta-analysis for social science research.

[B11-behavsci-15-01078] Carsley D., Heath N. L. (2018). Effectiveness of mindfulness-based colouring for test anxiety in adolescents. School Psychology International.

[B12-behavsci-15-01078] Carsley D., Heath N. L. (2019). Evaluating the effectiveness of a mindfulness coloring activity for test anxiety in children. The Journal of Educational Research.

[B13-behavsci-15-01078] Carsley D., Heath N. L., Fajnerova S. (2015). Effectiveness of a classroom mindfulness coloring activity for test anxiety in children. Journal of Applied School Psychology.

[B14-behavsci-15-01078] Carsley D., Khoury B., Heath N. L. (2018). Effectiveness of mindfulness interventions for mental health in schools: A comprehensive meta-analysis. Mindfulness.

[B15-behavsci-15-01078] Cheshure A., Stanwood G. D., Van Lith T., Pickett S. M. (2023). Distinguishing difference through determining the mechanistic properties of mindfulness based art therapy. Current Research in Behavioral Sciences.

[B16-behavsci-15-01078] Coholic D. A. (2011). Exploring the feasibility and benefits of arts-based mindfulness-based practices with young people in need: Aiming to improve aspects of self-awareness and resilience. Child & youth care forum.

[B17-behavsci-15-01078] Collaborators G. M. D. (2022). Global, regional, and national burden of 12 mental disorders in 204 countries and territories, 1990–2019: A systematic analysis for the Global Burden of Disease Study 2019. The Lancet Psychiatry.

[B18-behavsci-15-01078] Craske M. G., Rauch S. L., Ursano R., Prenoveau J., Pine D. S., Zinbarg R. E. (2011). What is an anxiety disorder?. Focus.

[B19-behavsci-15-01078] Cross G., Brown P. M. (2019). A comparison of the positive effects of structured and nonstructured art activities. Art Therapy.

[B20-behavsci-15-01078] Curry N. A., Kasser T. (2005). Can coloring mandalas reduce anxiety?. Art Therapy.

[B21-behavsci-15-01078] Dias Lopes L. F., Chaves B. M., Fabrício A., Porto A., Machado de Almeida D., Obregon S. L., Pimentel Lima M., Vieira da Silva W., Camargo M. E., da Veiga C. P. (2020). Analysis of well-being and anxiety among university students. International Journal of Environmental Research and Public Health.

[B22-behavsci-15-01078] Duong K., Stargell N. A., Mauk G. W. (2018). Effectiveness of coloring mandala designs to reduce anxiety in graduate counseling students. Journal of Creativity in Mental Health.

[B23-behavsci-15-01078] Egger M., Smith G. D., Schneider M., Minder C. (1997). Bias in meta-analysis detected by a simple, graphical test. BMJ.

[B24-behavsci-15-01078] Fulambarkar N., Seo B., Testerman A., Rees M., Bausback K., Bunge E. (2023). Meta-analysis on mindfulness-based interventions for adolescents’ stress, depression, and anxiety in school settings: A cautionary tale. Child and Adolescent Mental Health.

[B25-behavsci-15-01078] Galla B. (2024). How motivation restricts the scalability of universal school-based mindfulness interventions for adolescents. Child Development Perspectives.

[B26-behavsci-15-01078] Garmon B., Philbrick J., Daniel Becker M., John Schorling M., Padrick M., Goodman M. (2014). Mindfulness-based stress reduction for chronic pain: A systematic review. Journal of Pain Management.

[B27-behavsci-15-01078] Gotink R. A., Meijboom R., Vernooij M. W., Smits M., Hunink M. M. (2016). 8-week mindfulness based stress reduction induces brain changes similar to traditional long-term meditation practice—A systematic review. Brain and Cognition.

[B28-behavsci-15-01078] Gouda S., Luong M. T., Schmidt S., Bauer J. (2016). Students and teachers benefit from mindfulness-based stress reduction in a school-embedded pilot study. Frontiers in Psychology.

[B29-behavsci-15-01078] Gould L. F., Dariotis J. K., Mendelson T., Greenberg M. T. (2012). A school-based mindfulness intervention for urban youth: Exploring moderators of intervention effects. Journal of Community Psychology.

[B30-behavsci-15-01078] Grecucci A., Pappaianni E., Siugzdaite R., Theuninck A., Job R. (2015). Mindful emotion regulation: Exploring the neurocognitive mechanisms behind mindfulness. BioMed Research International.

[B31-behavsci-15-01078] Hedges L. V., Olkin I. (2014). Statistical methods for meta-analysis.

[B32-behavsci-15-01078] Higgins J. P., Thompson S. G. (2002). Quantifying heterogeneity in a meta-analysis. Statistics in Medicine.

[B33-behavsci-15-01078] Hilton L., Hempel S., Ewing B. A., Apaydin E., Xenakis L., Newberry S., Colaiaco B., Maher A. R., Shanman R. M., Sorbero M. E. (2017). Mindfulness meditation for chronic pain: Systematic review and meta-analysis. Annals of Behavioral Medicine.

[B34-behavsci-15-01078] Hofmann S. G., Sawyer A. T., Witt A. A., Oh D. (2010). The effect of mindfulness-based therapy on anxiety and depression: A meta-analytic review. Journal of Consulting and Clinical Psychology.

[B35-behavsci-15-01078] Holt N. J., Furbert L., Sweetingham E. (2019). Cognitive and affective benefits of coloring: Two randomized controlled crossover studies. Art Therapy.

[B36-behavsci-15-01078] Kabat-Zinn J. (2003). Mindfulness-based interventions in context: Past, present, and future. Clinical Psychology: Science and Practice.

[B37-behavsci-15-01078] Kabat-Zinn J. (2013a). Full catastrophe living, revised edition: How to cope with stress, pain and illness using mindfulness meditation.

[B38-behavsci-15-01078] Kabat-Zinn J. (2013b). Some reflections on the origins of MBSR, skillful means, and the trouble with maps. Mindfulness.

[B39-behavsci-15-01078] Kmet L. M., Cook L. S., Lee R. C. (2004). Standard quality assessment criteria for evaluating primary research papers from a variety of fields.

[B40-behavsci-15-01078] Lal D. M., Vinod Kumar S. (2024). Mindfulness-based intervention on psychological factors among students: A meta-analytic study. Journal of Rational-Emotive & Cognitive-Behavior Therapy.

[B41-behavsci-15-01078] Ledesma D., Kumano H. (2009). Mindfulness-based stress reduction and cancer: A meta-analysis. Psycho-Oncology: Journal of the Psychological, Social and Behavioral Dimensions of Cancer.

[B42-behavsci-15-01078] Lee S.-L. (2018). Why color mandalas? A study of anxiety-reducing mechanisms. Art Therapy.

[B43-behavsci-15-01078] Lensen J., Stoltz S., Kleinjan M., Speckens A., Kraiss J., Scholte R. (2021). Mindfulness-based stress reduction intervention for elementary school teachers: A mixed method study. Trials.

[B44-behavsci-15-01078] Léger-Goodes T., Malboeuf-Hurtubise C., Herba C. M., Taylor G., Mageau G. A., Chadi N., Lefrançois D. (2023). Videoconference-led art-based interventions for children during COVID-19: Comparing mindful mandala and emotion-based drawings. Mental Health Science.

[B45-behavsci-15-01078] Light R. J., Pillemer D. B. (1984). Summing up: The science of reviewing research.

[B46-behavsci-15-01078] Lipsey M. W., Wilson D. B. (2001). Practical meta-analysis.

[B47-behavsci-15-01078] Malboeuf-Hurtubise C., Léger-Goodes T., Mageau G. A., Taylor G., Herba C. M., Chadi N., Lefrançois D. (2021). Online art therapy in elementary schools during COVID-19: Results from a randomized cluster pilot and feasibility study and impact on mental health. Child and Adolescent Psychiatry and Mental Health.

[B48-behavsci-15-01078] Mantzios M., Giannou K. (2018). When did coloring books become mindful? Exploring the effectiveness of a novel method of mindfulness-guided instructions for coloring books to increase mindfulness and decrease anxiety. Frontiers in Psychology.

[B49-behavsci-15-01078] Noor S. M., Saleem T., Azmat J., Arouj K. (2017). Mandala-coloring as a therapeutic intervention for anxiety reduction in university students. Pakistan Armed Forces Medical Journal.

[B50-behavsci-15-01078] Öhman A. (2008). Fear and anxiety. Handbook of emotions.

[B51-behavsci-15-01078] Pearcey S., Gordon K., Chakrabarti B., Dodd H., Halldorsson B., Creswell C. (2021). Research Review: The relationship between social anxiety and social cognition in children and adolescents: A systematic review and meta-analysis. Journal of Child Psychology and Psychiatry.

[B52-behavsci-15-01078] Rapgay L., Bystrisky A. (2009). Classical mindfulness: An introduction to its theory and practice for clinical application. Annals of the New York Academy of Sciences.

[B53-behavsci-15-01078] Ross S. M., Morrison G. R. (2013). Experimental research methods. Handbook of research on educational communications and technology.

[B54-behavsci-15-01078] Russell G., Topham P. (2012). The impact of social anxiety on student learning and well-being in higher education. Journal of Mental Health.

[B55-behavsci-15-01078] Santomauro D. F., Herrera A. M. M., Shadid J., Zheng P., Ashbaugh C., Pigott D. M., Abbafati C., Adolph C., Amlag J. O., Aravkin A. Y. (2021). Global prevalence and burden of depressive and anxiety disorders in 204 countries and territories in 2020 due to the COVID-19 pandemic. The Lancet.

[B56-behavsci-15-01078] Sari Ozturk C., Kilicarslan Toruner E. (2022). The effect of mindfulness-based mandala activity on anxiety and spiritual well-being levels of senior nursing students: A randomized controlled study. Perspectives in Psychiatric Care.

[B57-behavsci-15-01078] Schanche E., Vøllestad J., Binder P.-E., Hjeltnes A., Dundas I., Nielsen G. H. (2020). Participant experiences of change in mindfulness-based stress reduction for anxiety disorders. International Journal of Qualitative Studies on Health and Well-Being.

[B58-behavsci-15-01078] Schuman-Olivier Z., Trombka M., Lovas D. A., Brewer J. A., Vago D. R., Gawande R., Dunne J. P., Lazar S. W., Loucks E. B., Fulwiler C. (2020). Mindfulness and behavior change. Harvard Review of Psychiatry.

[B59-behavsci-15-01078] Spielberger C. D. (1983). State-trait anxiety inventory for adults.

[B60-behavsci-15-01078] Spitzer R. L., Kroenke K., Williams J. B., Löwe B. (2006). A brief measure for assessing generalized anxiety disorder: The GAD-7. Archives of Internal Medicine.

[B61-behavsci-15-01078] Strohmaier S., Jones F. W., Cane J. E. (2021). Effects of length of mindfulness practice on mindfulness, depression, anxiety, and stress: A randomized controlled experiment. Mindfulness.

[B62-behavsci-15-01078] Thalheimer W., Cook S. (2002). How to calculate effect sizes from published research: A simplified methodology. Work-Learning Research.

[B63-behavsci-15-01078] Twenge J. M. (2000). The age of anxiety? The birth cohort change in anxiety and neuroticism, 1952–1993. Journal of Personality and Social Psychology.

[B64-behavsci-15-01078] Vago D. R., Silbersweig D. A. (2012). Self-awareness, self-regulation, and self-transcendence (S-ART): A framework for understanding the neurobiological mechanisms of mindfulness. Frontiers in Human Neuroscience.

[B65-behavsci-15-01078] Van Der Vennet R., Serice S. (2012). Can coloring mandalas reduce anxiety? A replication study. Art Therapy.

[B66-behavsci-15-01078] Viechtbauer W. (2007). Confidence intervals for the amount of heterogeneity in meta-analysis. Statistics in Medicine.

[B67-behavsci-15-01078] Williams J. M. G. (2008). Mindfulness, depression and modes of mind. Cognitive Therapy and Research.

[B68-behavsci-15-01078] Yang X., Fang Y., Chen H., Zhang T., Yin X., Man J., Yang L., Lu M. (2021). Global, regional and national burden of anxiety disorders from 1990 to 2019: Results from the Global Burden of Disease Study 2019. Epidemiology and Psychiatric Sciences.

[B69-behavsci-15-01078] Yılmazer E., Hamamci Z., Türk F. (2024). Effects of mindfulness on test anxiety: A meta-analysis. Frontiers in Psychology.

[B70-behavsci-15-01078] Zhang Y., Bao X., Yan J., Miao H., Guo C. (2021). Anxiety and depression in Chinese students during the COVID-19 pandemic: A meta-analysis. Frontiers in Public Health.

